# The Peroxisome Proliferator-Activated Receptor Gamma System Regulates Ultraviolet B-Induced Prostaglandin E_2_ Production in Human Epidermal Keratinocytes

**DOI:** 10.1155/2010/467053

**Published:** 2010-05-19

**Authors:** Raymond L. Konger, Kellie Clay Martel, Danielle Jernigan, Qiwei Zhang, Jeffrey B. Travers

**Affiliations:** ^1^Department of Pathology & Laboratory Medicine, Indiana University School of Medicine, Indianapolis, IN 46202, USA; ^2^Department of Dermatology, Indiana University School of Medicine, Indianapolis, IN 46202, USA; ^3^Department of Pediatrics and the H. B. Wells Center for Pediatric Research, Indiana University School of Medicine, Indianapolis, IN 46202, USA; ^4^Department of Pharmacology and Toxicology, Indiana University School of Medicine, Indianapolis, IN 46202, USA; ^5^Department of Dermatology, Richard L. Roudebush VA Medical Center, Indianapolis, IN 46202, USA

## Abstract

Studies using PPAR*γ* agonists in mouse skin have suggested that peroxisome proliferator-activated receptor gamma (PPAR*γ*) is irrelevant to cutaneous photobiology. However, in several epithelial cell lines, ultraviolet B (UVB) has been shown to induce the nonenzymatic production of oxidized phospholipids that act as PPAR*γ* agonists. UVB is also a potent inducer of prostaglandin E_2_ (PGE_2_) production and COX-2 expression in keratinocytes and PPAR*γ* is coupled to increased PGE_2_ production in other cell lines. In this current study, we demonstrate that PPAR*γ* agonists, but not PPAR*α* or PPAR*β*/*δ* agonists, induce PGE_2_ production and COX-2 expression in primary human keratinocytes (PHKs). Importantly, PPAR*γ* agonist-induced COX-2 expression and PGE_2_ production were partially inhibited by the PPAR*γ* antagonist, GW9662, indicating that both PPAR*γ*-dependent and -independent pathways are likely involved. GW9662 also suppressed UVB and *tert*-butylhydroperoxide- (TBH-) induced PGE_2_ production in PHKs and intact human epidermis and partially inhibited UVB-induced COX-2 expression in PHKs. These findings provide evidence that PPAR*γ* is relevant to cutaneous photobiology in human epidermis.

## 1. Introduction

Peroxisome proliferator-activated receptors (PPARs) are ligand-activated nuclear transcription factors that were initially identified as being crucial for regulating the formation of intracellular organelles, called peroxisomes, that are involved in lipid metabolism (reviewed in [1]). Three different PPARs subtypes have been cloned (*α*, *β*/*δ*, and *γ*) that differ in ligand specificity, tissue expression, and transcriptional targets. All three PPARs require heterodimerization with the retinoid X receptors (RXRs) for transcriptional activity. PPAR:RXR heterodimers induce target gene transcription by binding to specific peroxisome proliferators response elements (PPREs) in the promoter region of target genes [1]. Importantly, PPAR*α*, PPAR*β*/*δ*, and PPAR*γ* are all expressed in keratinocytes and in human and rodent epidermis [1–3]. We have recently demonstrated that PPAR*γ* is expressed in adult primary human keratinocytes and three different immortalized or malignant human keratinocyte cell lines (A431, HaCaT, and KB cells) [4, 5]. In KB epidermoid carcinoma cells and SZ95 sebocytes cells, we have also demonstrated that oxidative stress, including ultraviolet B irradiation, results in the production of oxidized lipid species with potent PPAR*γ* ligand activity [4, 5]. 

Natural PPAR*γ* ligands include metabolites of both the cyclooxygenase (COX) and lipoxygenase pathways, including the cyclopentanone prostaglandin, 15-deoxy-Δ^12,14^-prostaglandin J_2_, and 13-hydroxyoctadecadienoic acid (13-HODE) (reviewed in [2]). However, these compounds are relatively low-affinity ligands that also exhibit PPAR*γ*-independent functions [6]. The most potent natural ligands for PPAR*γ* have been shown to be oxidized alkyl phospholipids. This includes 1-hexadecyl 2-azelaoyl phosphatidylcholine (azPC), a nonenzymatically oxidized alkyl glycerophosphocholine first discovered associated with oxidized low-density lipoprotein [7]. Importantly, azPC has been shown to be produced following UVB irradiation [4]. In addition, the thiazolidinedione (TZD) compounds, troglitazone, ciglitazone, rosiglitazone, and pioglitazone, are synthetic PPAR*γ* agonists that are widely used in the treatment of type II diabetes. However, synthetic TZD PPAR*γ* agonists have also been shown to exhibit PPAR*γ*-independent effects [8–10]. This underscores the importance of using appropriate controls, such as the PPAR*γ*-selective antagonist, GW9662, to verify PPAR*γ*-specific results when using these agonists [11]. 

While it is clear that UVB and other oxidative stressors can result in PPAR*γ* ligand production, a previous study using exogenous PPAR*γ* agonists failed to demonstrate any effect on either chemical carcinogenesis or UVB-induced skin cancer formation [12]. These negative findings raise doubts concerning the relevance of PPAR*γ* to cutaneous photobiology. Yet, it should be noted that mice with heterozygous germline deletion of PPAR*γ* or mice with epidermal-specific loss of PPAR*γ* exhibit an increase in chemical carcinogen-induced skin tumors [13, 14]. This suggests the possibility that loss of function models, such as the use of PPAR*γ* antagonists rather than agonists, might be more informative for studies designed to examine the role of PPAR*γ* in photobiology. Given that we have already demonstrated that UVB induces PPAR*γ* ligand formation, we hypothesized that the lack of effect of exogenous PPAR*γ* ligands on photocarcinogenesis could be explained by the fact that PPAR*γ* is already engaged by UVB-induced ligand production. Inasmuch as our previous studies using cell lines indicate that PPAR*γ* is coupled to epithelial COX-2 expression and PGE_2_ production, we therefore utilized the PPAR*γ* antagonist, GW9662, to determine whether PPAR*γ* is involved in regulating UVB-induced COX-2 expression and PGE_2_ production in primary human keratinocytes and intact human epidermal explants. The results of the present studies indicate that PPAR*γ* is functionally coupled to a readily measured photobiological response in human primary epidermal keratinocytes and is therefore relevant to cutaneous photobiology.

## 2. Materials and Methods

### 2.1. Materials

 Ciglitazone, GW501516, and WY-14,643 were obtained from Alexis Biochemicals (San Diego, CA). AzPC (1-O-Hexadecyl-2-Azelaoyl-sn-Glycero-3-Phosphocholine) was purchased from Avanti Polar Lipids (Alabaster, AL). The specific PPAR*γ* antagonist, GW9662, was obtained from Cayman Chemical (Ann Arbor, MI). The selective COX-2 inhibitor, NS398, was obtained from Sigma-Aldrich (St. Louis, MO). All other reagents were obtained from Sigma-Aldrich unless otherwise noted.

### 2.2. Cell Culture

 Adult primary human keratinocytes (PHKs) were prepared from discarded epidermis that was obtained from reductive mammoplasties and panniculectomies as previously described [15]. Telomerase-immortalized primary human keratinocytes (N/TERT-1) were obtained from Dr. Rheinwald (Department of Medicine and Harvard Skin Disease Research Center, Brigham, and Women's Hospital, Boston, MA) [16]. PHKs and N/TERT-1 cells were cultured on tissue culture plastic or wells that were precoated with type I collagen. PHKs and N/TERT-1 cells were grown in serum-free media (Keratinocyte serum-free media, K-SFM; Gibco Invitrogen, Carlsbad, CA). Media were supplemented with 40 IU per mL penicillin, 40 *μ*g per mL streptomycin, and 0.1 *μ*g per mL amphotericin B. The cells were cultured in 95% air and 5% CO_2_ at 37°C. All studies were done using PHKs or N/TERT-1 cells that were plated at sufficient density to achieve approximately 70%–80% confluence prior to experimental manipulation. All studies with human skin have been approved by the Indiana University-Purdue University at Indianapolis Institutional Review Boards using the Declaration of Helsinki Principles.

### 2.3. Immunoblotting

 Immunoblotting for PPAR*γ* was done using mouse monoclonal anti-PPAR*γ* antibody (clone E8; Santa Cruz Biotechnology, Santa Cruz, CA), essentially as described in [4]. The specificity of this antibody for PPAR*γ* has previously been demonstrated in wild-type versus tissue-specific PPAR*γ* knockout mice [17].

### 2.4. Ultraviolet B Irradiation of Cultured Keratinocytes

 For UVB irradiation studies, a Philips F20T12/UV-B lamp (270–390 nm), containing 2.6% UVC, 43.6% UVB, and 53.8% UVA, was utilized. The UVB dose was measured using an IL1700 radiometer and a SED240 UVB detector (International Light, Newburyport, MA). All irradiations were performed at a distance of 8 cm from the UVB light source. All irradiations were done on PHKs grown in 24-well plates. For GW9662- and NS398-treated cells, media containing vehicle, 1 *μ*M GW9662, or 10 *μ*M NS398 were added either 1 hour (GW9662) or 30 minutes (NS398) prior to UVB irradiation. The cells were then washed twice with PBS, and then the cells were irradiated with 600 J/m^2^ UVB. DMEM containing 10% FBS was then added containing vehicle, GW9662, or NS398. After eight hours at 37°C, the culture supernatants were removed for PGE_2_ quantitation, the cell monolayer was trypsinized, and the cells were counted. PGE_2_ was then normalized to cell count.

### 2.5. Tert-Butylhydroperoxide (TBH) Studies in PHKs In Vitro

 For TBH studies, cells were plated onto 24-well plates in high-calcium DMEM containing 10% fetal bovine serum. The cells were then pretreated with GW9662 or NS398 as detailed in the previous section prior to TBH addition. Culture supernatants were then collected after an eight-hour incubation at 37°C.

### 2.6. PPAR Agonist Studies

 For PPAR agonist studies, agonists were added in low-calcium K-SFM media or in K-SFM media supplemented with 1 mM calcium and incubated for 24 hours at 37°C. To assess PPAR*γ*-dependent induction of PGE_2_, GW9662 (1 *μ*M) was added 1 hour prior to agonist addition. PGE_2_ was then quantitated in tissue culture media by EIA and normalized to either cell count or total cellular protein (BCA assay; Pierce Biotechnology, Rockford, IL).

### 2.7. RNA Isolation and COX-2 Quantitative RT-PCR

 PHKs were treated with vehicle and ciglitazone (with and without GW9662) as detailed in the previous section. At 2, 4, 8, and 24 hours after the addition of reagents, the cell monolayers were processed for RNA isolation using an RNeasy kit (Qiagen) according to the manufacturer's protocol. Following first-strand DNA synthesis, quantitative RT-PCR (qRT-PCR) was performed using primers specific to human COX-2 and 18S rRNA with a Cepheid Smart Cycler real-time PCR instrument (Fisher Scientific, Pittsburgh, PA). COX-2 and 18S qRT-PCR were performed as previously described [5]. COX-2 results were then normalized to 18S using the ΔΔCt method [18].

### 2.8. Human Epidermal Explant Preparation and UVB Irradiation

 Adult human epidermis obtained from panniculectomies was obtained postoperatively. Subdermal fat and a portion of the lower dermis were immediately removed using a scalpel blade. The epidermis was then cut into small (approx. 8 × 8 mm) sections, weighed, and then cultured in 12-well plates submerged in Keratinocyte-SFM media for 48–72 hours. The explants were then pretreated with vehicle, 1 *μ*M GW9662 (1 hour), or 10 *μ*M NS398 (30 minutes) prior to UV treatment. After pretreatment, the explants were washed twice with PBS and a minimal amount of PBS was added to cover the explants to maintain hydration. The explants were then irradiated with 1,800 J/m^2^ of UVB light. Control cells received no UVB light. The explants were then submerged in serum-free DMEM containing vehicle, 1 *μ*M GW9662, or 10 *μ*M NS398 and cultured for 8 hours at 37°C. At this time the media were removed for PGE_2_ quantitation. All PGE_2_ levels were then normalized to tissue weight.

### 2.9. COX-2 Immunoblot

Second-passage PHKs grown on 6-well plates to near confluence were treated with 1 *μ*M GW9662 or vehicle 1 hour prior to washing the wells with HBSS, then irradiating the cells with 300 J/m^2^ of UVB. The HBSS was then replaced with media containing vehicle or 1 *μ*M GW9662, and the cells were processed 20 hours later in RIPA buffer supplemented 1 : 100 with a protease inhibitor cocktail (Sigma-Aldrich). Protein was quantitated using a DC protein assay, and equal amounts of total protein (50 *μ*g/lane) were then separated on 7.5% SDS-PAGE gels. Rabbit polyclonal anti-COX-2 (Cayman Chemical) diluted 1 : 1000 in TBS with 0.1% Tween 20 and 2% Blotto was added and incubated overnight at 4°C in 2% Blotto. Goat anti-rabbit HRP conjugate (1 : 10,000; Source) in 5% Blotto was applied and the immunoreactive bands were detected enhanced chemiluminescence. The exposed films were developed and scanned and band intensity was determined using NIH Image J software. The band intensity of the COX-2 band at 72 kDa was normalized to that of an invariant non-specific high-molecular-weight band (>180 kDa) observed in all lanes.

### 2.10. PGE_2_ Quantitation

PGE_2_ was quantitated as previously described in culture media using a commercial PGE_2_ EIA kit (Cayman Chemical, Ann Arbor, MI) [4].

## 3. Results and Discussion

### 3.1. Primary Human Keratinocytes Express PPAR*γ*

**Figure 1:**
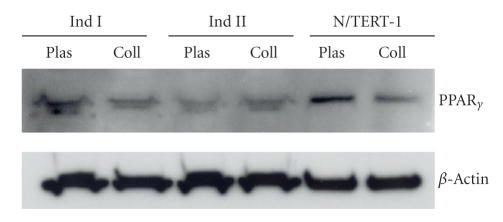
PPAR*γ* expression by immunoblot in PHKs. 40 *μ*g cellular protein isolated from PHKs from two different individuals was grown on tissue culture plastic coated with (Coll) and without (Plas) type I rat tail collagen. Protein from telomerase-immortalized primary human keratinocytes (N/TERT-1) was also utilized. In all cases, the proteins were separated on a 10% SDS-PAGE and PPAR*γ* protein was detected using a monoclonal anti-PPAR*γ* antibody. Loading was verified by stripping the blots and performing an immunoblot for *β*-Actin.

 Studies in mouse skin have shown that PPAR*γ* is expressed at low levels and may be functionally irrelevant in mouse keratinocytes [8, 12]. We therefore first verified that PPAR*γ* is expressed in the PHKs from several different individuals under several different culture conditions (culture on plastic or type I collagen) (Figure 1). To exclude the possibility that the PPAR*γ* seen in these primary cultures was derived from other cell types, immunoblotting for PPAR*γ* was also done on telomerase-immortalized human keratinocytes (N/TERT-1) [16]. This data supports previous reports that PHKs express mRNA and protein for PPAR*γ* [19–21].

### 3.2. PPAR*γ* Agonists, but not PPAR*α* and PPAR*β*/*δ* Agonists, Stimulate PGE_2_ Formation in PHKs through Both PPAR*γ*-Dependent and -Independent Mechanisms

**Figure 2:**
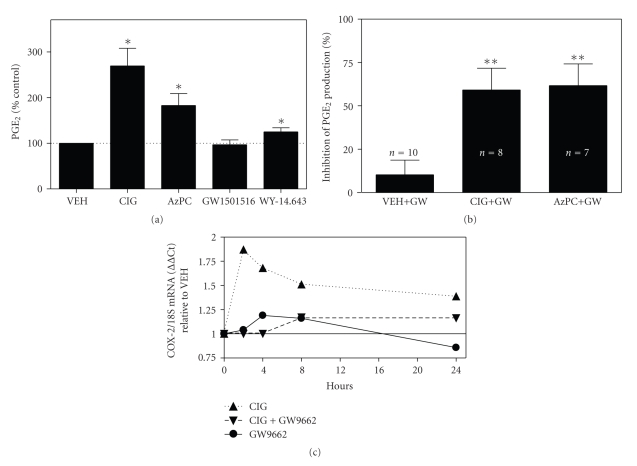
PPAR*γ* agonists, but not PPAR*α* or *β*/*δ* agonists, induce PGE_2_ production in PHKs; PPAR*γ* agonist-induced PGE_2_ production and COX-2 mRNA expression are inhibited by GW9662. (a) PPAR*γ* agonists, but not PPAR*α* or *β*/*δ* agonists, induce PGE_2_ production in PHKs. PHKs were treated for 24 hours with vehicle (CONT), 5 *μ*M of the PPAR*γ* agonist, ciglitazone (CIG), 1 *μ*M of the endogenous PPAR*γ* agonist, azPC, a PPAR*α* agonist (WY-14,643, 1 *μ*M), or a PPAR*β*/*δ* agonist (GW501516, 500 nM). The media were then removed and PGE_2_ was quantitated in the culture media using a commercial PGE_2_ EIA kit. Values represent the mean ± SEM of PGE_2_ levels as a percent of control levels (*N* = 5 experiments done in triplicate). *P < .05, one sample *t*-test**. **(b) GW9662 inhibits PPAR*γ* agonist-induced PGE_2_ production in PHKs but has no significant effect on vehicle-treated cells. PHKs were pretreated for 1 hour with 1 *μ*M GW9662 prior to addition of vehicle (VEH), ciglitazone (5 *μ*M), or azPC (1 *μ*M) for 24 hours. PGE_2_ was then quantitated in tissue culture supernatants. The results shown represent the percent inhibition of vehicle or agonist-induced PGE_2_ formation by GW9662. *Mean and SEM*. **P < .01, one-sample *t*-test. (c) GW9662 treatment inhibits ciglitazone-induced COX-2 mRNA expression. PHKs were treated with vehicle and ciglitazone, with and without GW9662, as detailed in Figure 2(b) above. At 2, 4, 8, and 24 hours, total RNA was isolated and quantitative RT-PCR was performed for COX-2 mRNA and 18S rRNA expression. The results are shown as COX-2 expression normalized to 18S and expressed as a fold change relative to VEH control (assigned a value of 1 for all time points). When data for all time points was analyzed, only ciglitazone-treated cells exhibited a significant induction in COX-2 expression (one-sample *t*-test, *P* = .0101).

 In SZ95 sebocytes, we have previously demonstrated that GW9662 was effective in blocking PGE_2_ production elicited by the synthetic PPAR*γ* agonist ciglitazone [5]. We therefore examined whether ciglitazone could also induce PGE_2_ formation in PHKs. Given that a TZD type of PPAR*γ* agonist has been reported to induce COX-2 expression independent of PPAR*γ* in a mouse keratinocyte cell line [22], we also examined whether the endogenous PPAR*γ* ligand, azPC, could also induce PGE_2_ production in PHKs. Finally, the ability of PPAR*α* or PPAR*β*/*δ* to regulate PGE_2_ production in PHKs was also examined. In Figure 2(a), we demonstrate that treatment of PHKs with the PPAR*γ* agonists, ciglitazone, and azPC results in a significant increase in PGE_2_ production. The PPAR*α* agonist, WY-14,643, resulted in only a slight, but reproducible, increase in PGE_2_ production. Treatment with a PPAR*β*/*δ* agonist (GW501516, 500 nM) did not alter PGE_2_ production. It should be noted that NS398 significantly blocked both basal and PPAR*γ* agonist-induced changes in PGE_2_ production. As a percent of the vehicle control-treated cells, PGE_2_ levels were 8.1 ± 7.4% (*n* = 4), 11.2 ± 12.5% (*n* = 4), and 12.2 ± 8.8% (*n* = 3) for NS398 treatment alone, ciglitazone + NS398, and azPC + NS398-treated cells, respectively.

The above studies suggest that PPAR*γ*, but not PPAR*α* or PPAR*β*/*δ*, is coupled to PGE_2_ production in PHKs. To determine whether ciglitazone- and azPC-dependent PGE_2_ production was specific to PPAR*γ*, we next examined the ability of GW9662 to block PPAR*γ* agonist-induced PGE_2_ production. As shown in Figure 2(b), pretreatment of PHKs with 1 *μ*M GW9662 resulted in a significant inhibition of both ciglitazone- and azPC-induced PGE_2_ production. However, this inhibition was not complete: GW9662 inhibited ciglitazone- and azPC-induced PGE_2_ production by 59% and 62%, respectively. In contrast, GW9662 had no significant effect on vehicle-treated keratinocytes. Inasmuch as this same dose of GW9662 completely inhibited ciglitazone-induced PGE_2_ formation in SZ95 sebocytes [5], this data suggests that PGE_2_ production induced by azPC and ciglitazone occurs through both PPAR*γ*-dependent and -independent mechanisms in PHKs.

As noted above, the ability of NS398 to inhibit basal and PPAR*γ* agonist-induced PGE_2_ production suggests that PPAR*γ* agonists induce expression of COX-2. We have previously demonstrated that PPAR*γ* is coupled to UVB- and tert-butylhydroperoxide-mediated COX-2 expression in SZ95 sebocytes. This idea is supported by studies in SZ95 sebocytes in which the PPAR*γ* antagonist GW9662 completely blocks PGE_2_ formation induced by ciglitazone, UVB, and TBH [5]. Moreover, the COX-2 promoter is known to contain putative peroxisome proliferators response elements (PPREs) [23]. Thus, we next examined the ability of ciglitazone to induce COX-2 expression in PHKs. In Figure 2(c), we show that COX-2 mRNA expression is induced from 2 to 24 hours after ciglitizone addition. Moreover, GW9662 treatment suppressed COX-2 induction.

### 3.3. PPAR*γ* Mediates UVB and Oxidative Stressor-Induced PGE_2_ Production

**Figure 3:**
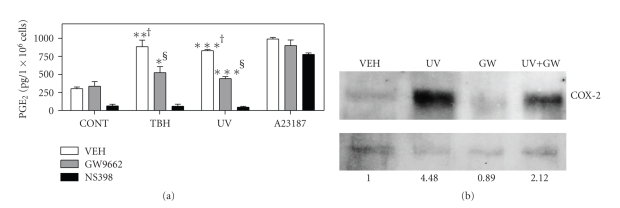
The specific PPAR*γ* antagonist GW9662 blocks UVB- and TBH-induced PGE_2_ production and UVB-induced COX-2 protein expression. (a) GW9662 inhibits UVB- and TBH-induced PGE_2_ production in PHKs. PHKs were pretreated with vehicle control (VEH), GW9662 (1 *μ*M) for 1 hour, or the COX-2 inhibitor NS398 (10 *μ*M) for 30 minutes, then treated with 10 *μ*M *tert*-butylhydroperoxide (TBH), 500 nM calcium ionophore A23187, or exposed to 600 J/m^2^ UVB irradiation (UV), respectively. PGE_2_ production was assayed in the tissue culture supernatants after 8 hours and was normalized to tissue weight. The values of PGE_2_ expression shown are mean ± SEM and are representative of three experiments (*P < .05, **P < .01, ***P < .001 by *t*-test compared with nonirradiated vehicle control †, or from the respective UV- or TBH-treated cells without GW9662 pretreatment §). (b) GW9662 inhibits UVB-induced COX-2 expression by immunoblot. PHKs were pretreated with vehicle (VEH) or 1 *μ*M GW9662 (GW) 1 hour prior to irradiation with 300 J/m^2^ UVB (UV). After 20 hours, total cellular protein was isolated and immunoblots were performed for COX-2 expression (a) as detailed in the methods section. (b) shows a high MW nonspecific invariant band used to normalize for COX-2 expression. After film exposure, band intensities were assessed using NIH Image J. Normalized band intensities are shown at the bottom as a relative increase from VEH control cells.

 We have previously demonstrated that UVB exposure activates PPAR*γ* via the production of oxidized lipid species in KB epidermal carcinoma cells and SZ95 sebocytes [4, 5]. Moreover, UVB-induced PPAR*γ* activation is necessary for optimal UVB-mediated COX-2 expression or PGE_2_ production in both KB cells and SZ95 sebocytes [4, 5]. Finally, the lipid soluble oxidant, TBH, also induces COX-2 and PGE_2_ production in SZ95 sebocytes through a PPAR*γ*-dependent mechanism [5]. We therefore sought to establish the importance of PPAR*γ* in photobiology by demonstrating that PPAR*γ* antagonism could alter UVB-mediated induction of PGE_2_ production in PHKs. As shown in Figure 3, both UVB and the lipid soluble oxidant TBH were capable of inducing a marked increase in PGE_2_ production in PHKs. Importantly, pretreatment with the PPAR*γ*-specific antagonist, GW9662, blocked 68% of the TBH and 80% of the UVB-mediated increases in PGE_2_. The ability of the selective COX-2 inhibitor, NS398, to inhibit PGE_2_ formation was utilized as a negative control. As expected, NS398 markedly suppressed basal and UVB-induced PGE_2_ production. The calcium ionophore, A23187, which is a potent activator of both COX-2 and upstream phospholipases, was utilized as an irrelevant positive control. As expected, GW9662 had no affect on ionophore-induced PGE_2_.

Finally, we examined the ability of GW9662 to inhibit UVB-induced COX-2 expression. In Figure 3(b), we show a representative immunoblot demonstrating that GW9662 strongly inhibits UVB-induced COX-2 expression (mean suppression of 61.5% in two separate experiments).

### 3.4. UVB-Induced PGE_2_ Production Occurs via PPAR*γ* in Human Epidermal Explants

**Figure 4:**
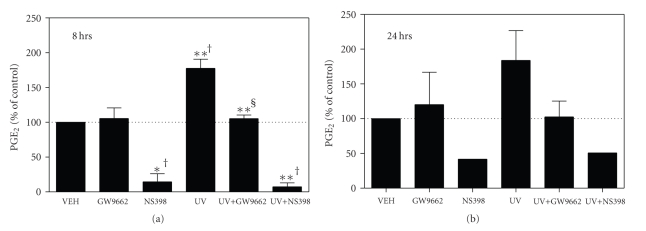
PPAR*γ* activation is a potent inducer of PGE_2_ production in human epidermal explants. Human epidermal explants were prepared as described in the methods section and placed into K-SFM media (Invitrogen). Prior to UVB (UV) irradiation, the explants were pretreated with 1 *μ*M GW9662 for 1 hour or 10 *μ*M NS398 for 30 minutes. The explants were then washed once in PBS and irradiated with 1800 J/m^2^ UVB. Media containing vehicle (CONT), 1 *μ*M GW9662, or 10 *μ*M NS398 were then added back and the explants were cultured for 8 hours (a) or 24 hours (b). Tissue culture supernatants were then removed for PGE_2_ quantitation by EIA. PGE_*2*_ results were normalized to tissue weight. Results are presented as a percent change from control PGE_2_ levels. Mean and SEM of 2–4 experiments were done in triplicate. (*P < .05, one-sample *t*-test compared with nonirradiated vehicle control †, or from UV-treated skin without GW9662 pretreatment §).

The above studies indicate that PPAR*γ* antagonism suppresses UVB- and TBH-induced PGE_2_ production and COX-2 expression in cultured PHKs in vitro. We next sought to determine whether PPAR*γ* antagonism could alter UVB-induced PGE_2_ formation in intact human skin ex vivo. In Figure 4(a), we show that skin explants exposed to 1,800 J/m2 of UVB exhibited a significant increase in PGE_2_ production at 8 hours. Importantly, UVB-induced PGE_2_ production was nearly completely blocked by pretreatment with 1 *μ*M GW9662. A similar induction of PGE_2_ production was observed 24 hours after UVB irradiation, which was also blocked by pretreatment with GW9662 (Figure 4(b)). Finally, as a negative control, we show that the COX-2 inhibitor, NS398, suppresses both control and irradiated PGE_2_ levels. These studies show that GW9662 is effective topically and also verifies the importance of PPAR*γ* in mediating a photobiological response in intact human skin.

## 4. Conclusions

Inasmuch as the epidermis is constantly exposed to ultraviolet light and other oxidative stressors, it is not surprising that epidermal cells would have developed intracellular mechanisms to detect and respond to these stresses. Among the many cellular signaling pathways that are induced by UV light, COX-2 induction and PGE_2_ production have been well described [5, 23, 24]. We have recently demonstrated that UVB induces PGE_2_ synthesis via the production of oxidative-stress-induced PPAR*γ*-specific ligands in other cell types [4, 5]. Thus, PPAR*γ* may serve as a cellular “signal transducer” that converts oxidative stress into cellular responses. In this study, we demonstrate that PPAR*γ* agonists, including ciglitazone and azPC, are capable of inducing PGE_2_ production in cultured PHKs. We then demonstrate that UVB- and TBH-induced PGE_2_ production in human epidermis ex vivo and cultured PHKs in vitro occurs via a PPAR*γ*-dependent mechanism. The role of COX-2 in PGE_2_ production in PHKs was verified by demonstrating that GW9662 inhibits ciglitazone-induced COX-2 mRNA expression as well as UVB-induced COX-2 protein expression. Collectively, these findings strongly suggest that PPAR*γ* acts as a photosensor (and likely an oxidative stress sensor) that acts to translate the insult into a biochemical signaling cascade.

Previous work has generated conflicting data regarding the functional relevance of PPAR*γ* in epidermal and keratinocyte biology. In mice, one group failed to observe PPAR*γ* expression in cultured SKH-1 hairless mouse keratinocytes by RT-PCR [8]. In yet another study, PPAR*γ* was observed to be expressed at the mRNA level in human primary keratinocytes, yet functional PPAR*γ* activity was not observed using a PPRE-luciferase reporter assay [19]. However, another group demonstrated that PPAR*γ* was expressed in keratinocytes isolated from SENCAR mice using immunoblot analysis [25]. Yet another study using PPAR*γ* conditional knockout mice demonstrated that PPAR*γ* is involved in epidermal differentiation [26]. In our studies, we demonstrate that PPAR*γ* is expressed at the protein level in primary human keratinocytes and that PPAR*γ* is coupled to UVB- and TBH-induced PGE_2_ production. Our findings that PPAR*γ* mediates UVB- and TBH-induced PGE_2_ production provide additional evidence that PPAR*γ* is functionally relevant to human cutaneous biology and, in particular, is relevant to human keratinocyte photobiology. This is particularly important given previous work by our group and others demonstrating that endogenous PPAR*γ* ligand formation is induced by oxidative stress, including UVB [4, 5, 27, 28].

While our studies indicate a clear role for PPAR*γ* in regulating UVB- and TBH-induced PGE_2_ production in PHKs, previous studies have shown that PPAR*α* and PPAR*β*/*δ* are also expressed in human epidermis and cultured keratinocytes [1, 20]. Though a PPAR*α* agonist induced a small amount of PGE_2_ formation in PHKs (Figure 2(a)), our data does not support a role for either PPAR*α* or PPAR*β*/*δ* as major mediators of increased PGE_2_ production in PHKs. 

Recent studies have demonstrated that mice with hemizygous germline loss of PPAR*γ* and epidermal-specific loss of PPAR*γ* exhibit increased susceptibility to cutaneous chemical carcinogenesis [13, 14]. This data indicates a possible role for PPAR*γ* as a tumor-suppressing agent in skin. Studies are currently underway to examine the potential role of PPAR*γ* in cutaneous photocarcinogenesis. It should be noted that recent studies by He et al. (2005) indicate that topical or systemic administration of TZD-type PPAR*γ* agonists have no significant effect on chemical (DMBA/phorbol ester) or UVB-mediated carcinogenesis [12]. There could be several explanations for this discrepancy. First, we and others have previously shown that UVB or other oxidative stressors induce PPAR*γ* ligand production from cellular glycerophosphocholines [4, 7]. Moreover, phorbol esters act to induce oxidative stress, including the production of oxidized lipids in epidermis [29, 30]. Thus, it is quite possible that PPAR*γ* receptors are already engaged by endogenous PPAR*γ* ligands produced through phospholipid oxidation, thus mitigating the effects of exogenous ligand. Alternatively, a followup study by He et al. (2006) indicated that TZD-type PPAR*γ* agonists induce COX-2 expression in a keratinocyte cell line lacking PPAR*γ* [22]. The authors speculate that this PPAR*γ*-independent production of protumorigenic COX-2 may counteract any tumor-suppressing function of PPAR*γ*. This PPAR*γ*-independent induction of COX-2 would also explain the results seen in Figures 2 and 3, in which GW9662 was unable to completely abolish PPAR*γ* agonist or UVB-induced PGE_2_ production and COX-2 expression. Finally, the differences could potentially be explained by variations in the murine genetic background or carcinogenesis protocol. 

In addition to a potential role for PPAR*γ* in cutaneous photocarcinogenesis, independent lines of evidence suggest that PPAR*γ* should be a focus for future studies examining its potential role as a mediator of phototherapeutic responses. Recently, PPAR*γ* agonists have been shown to be effective antipsoriatic agents by reducing the keratinocyte hyperplasia associated with psoriasis [31]. This is significant as UVB exposure is a well-known treatment option for patients with psoriasis. Since psoriasis is thought to occur as a result of deregulated T-cell function [32, 33], it is interesting that both PPAR*γ* [34–36] and PGE_2_ [37–39] act as T-cell and dendritic cell immunosuppressants and that topical application of PGE_2_ has been shown to result in clinical improvement of psoriatic lesions [40]. Given our findings that UVB activates keratinocyte PPAR*γ*, it is possible that some of the therapeutic benefits of UVB treatment may be due to PPAR*γ* activation.

In conclusion, we provide evidence that PPAR*γ* stimulates PGE_2_ formation in PHKs. Moreover, PPAR*γ* mediates UVB- and TBH-induced PGE_2_ production in human epidermis and primary human keratinocytes. This suggests that PPAR*γ* may play an important role in UVB- and oxidative-stress-induced changes in epidermal biology.
